# Accidental self-inoculation of a veterinary field worker with Calf Paratyphoid Vaccine

**DOI:** 10.4102/safp.v63i1.5400

**Published:** 2021-10-27

**Authors:** David E. Whittaker

**Affiliations:** 1 Private Practice, Family Physician, Cape Town, South Africa

A young B.Sc. graduate consulted a locum in a suburban general practice in Cape Town recently and said, ‘I’m worried that I may have cancer’. He was working as a veterinary field worker for a private organisation in Botswana that assists cattle farmers there to manage their herds more efficiently. A week before the consultation, he was inoculating a calf with Calf Paratyphoid Vaccine (CPV) when the calf jerked away from him and he accidentally injected the vaccine into his hand. The next day, he felt feverish, noticed swelling of his ‘glands’, and had pain and swelling in his testicles. Thoroughly frightened, he consulted a local doctor who examined him and performed an ultrasound scan (USS) of his scrotum. The doctor treated him for fever and pain and, noting swelling of the testicles on the scan said, ‘you may have cancer’. The young man could not recall the name of the doctor he had seen or what referral advice the doctor had given him, neither was it clear to him whether or not the doctor was trained in USS interpretation. Whatever else the doctor might have said to him, the young man could only remember that he might have cancer of the testicles. Highly alarmed, the young man phoned his parents who immediately arranged for him to fly home to Cape Town for review by their General Practitioner (GP). The GP being away, he saw the locum.

After listening to the young man’s unusual story and having examined him and found no testicular or other swelling, the locum assured him that he did not have cancer, and that the symptoms he had experienced, had most probably been caused by the systemic effects of the vaccine. The vaccine leaflet^1^ notes only that calves may have a raised temperature for 2–4 days after inoculation. His parents were only finally satisfied that their son did not have testicular cancer when a urologist assured them that he did not. Greatly relieved, the young man then went back to work in Botswana – he had been badly frightened and had learnt about the need to properly restrain calves for inoculation. His parents, however, were badly out of pocket by the costs of an airfare, four consultations and several expensive investigations.

This report of an uncommon but significant clinical event shows one of the hazards of veterinary field work, the harm that can be done to a patient by a doctor issuing a cancer warning from a single investigation, and the need to ensure that patients are clearly referred for further care when necessary. Always remembering that it is much easier to refer patients for further care in suburban Cape Town than it is in rural Botswana.

Accidental self-inoculation of veterinary workers with animal vaccines can lead to severe local injury especially when the inoculation is delivered through a pressurised device or when the vaccine has an oil-based adjuvant.^2^ Fortunately, this young man came to no permanent harm and was, as he said before he left, ‘very well protected against a disease I will never get!’
